# Microbial Keratinolysis: Eco-Friendly Valorisation of Keratinous Waste into Functional Peptides

**DOI:** 10.3390/microorganisms13102270

**Published:** 2025-09-27

**Authors:** Lindelwa Mpaka, Nonso E. Nnolim, Uchechukwu U. Nwodo

**Affiliations:** Patho-Biocatalysis Group (PBG), Department of Biotechnology and Biological Sciences, University of Fort Hare, Private Bag X1314, Alice 5700, South Africa; nnnolim@ufh.ac.za (N.E.N.); unwodo@ufh.ac.za (U.U.N.)

**Keywords:** keratinous biomass, biodegradation, keratinases, disulfide bonds, waste management

## Abstract

Keratinous wastes, generated from various industries such as poultry processing, slaughterhouses, and salons, accumulate in the environment due to their slow degradation caused by high disulfide cysteine bonds. Traditional methods of managing these wastes, including incineration, composting, open-air burning, and landfilling, have several disadvantages, such as environmental pollution, release of toxic compounds, and breeding of pathogenic and multidrug-resistant microorganisms. Microbial keratinases, produced by bacteria, fungi, and actinomycetes, offer an eco-friendly alternative for valorizing keratinous waste into valuable peptides and amino acids. The biodegradation of keratinous biomass involves four sequential steps: adhesion, colonization, production of keratinolytic enzymes, and breakdown of the keratin substrate. Optimization of culture conditions, such as pH, temperature, substrate concentration, and metal ions, can enhance keratinase production for industrial applications. Keratinases have multifaceted applications in various sectors, including cosmetics, organic fertilizers, leather treatment, animal feed, detergents, and pharmaceuticals. This review highlights the need to explore keratinolytic strains further and improve keratinase yields to develop sustainable solutions for keratinous waste management and generate value-added products, promoting a circular economy. The techno-economic considerations and current limitations in industrial-scale keratinase production are also discussed, emphasizing the importance of future research in this field.

## 1. Introduction

Keratinous biomass accumulates significantly in the environment due to its resistance to classical proteases, primarily because of a keratin protein [1,2]. Keratin is a protective structural protein found in vertebrates, including birds, humans, cattle, sheep, goats, and reptiles. It is present in nails, horns, skin, hooves, claws, feathers, beaks, and hair [3]. This protein enters the environment through keratinous waste from slaughterhouses, poultry processing, leather industries, barbershops, and the wool sector. These wastes tend to persist in the environment for extended periods. This longevity is attributed to the tightly packed disulfide bonds, hydrogen bonds, and hydrophobic interactions that contribute to the rigidity and durability of keratin [2,4].

The keratin protein is characterized into two main groups: the alpha helix and beta sheets and is further classified as soft or hard keratins based on cysteine content. Soft keratins are predicted to have less than 10% cysteine content; examples of soft keratins include skin and callus. In contrast, hard keratins contain cysteine levels ranging from 10% to 14%, and this type of keratin is found in nails, claws, hair, and hooves, playing a crucial role in protecting cells from external environmental stress [5,6]. The recalcitrance of this protein against classical protease activity has made it the third most abundant polymer, following chitin and cellulose [7,8].

The rise in population has pressured several industries, including the agro-industry, livestock industry, poultry processing, and textile industry, to meet the basic needs of this growing population. Consequently, these industries generate large amounts of keratinous waste, particularly the agro and livestock sectors and poultry processing [8,9,10]. The presence of keratin in these waste materials challenges waste management. Fortunately, these wastes are rich in diverse organic compounds, such as 2–5% sulphur, 15 to 18% nitrogen, 3.20% mineral elements, 1.27% fat, and 90% protein [1,11]. Thus, these keratinous wastes have potential for bioconversion into valuable products for diverse applications, including feed supplements for livestock, organic fertilizers for plant growth, feedstocks for bioenergy production, and bioactive compounds for skin care products [12,13,14,15]. However, despite their richness in several biological components, particularly keratin, recycling them remains complicated due to their recalcitrant tendencies conferred by their architectural disposition [1].

Various methods have been developed to tackle the challenge of recycling keratinous biomass, including both physical and chemical treatments. Nonetheless, these methods face considerable obstacles in recent times due to the huge energy requirement, ecological implications, and loss of labile products, producing poor-quality products [12,16,17,18]. Interestingly, recycling keratinous waste biomass into valuable products is essential, considering environmental regulations restricting waste disposal and the growing need for renewable resources valorization to promote a zero-waste economy [19,20].

Fortunately, a special class of proteases known as keratinases has significantly demonstrated its ability to dismember keratinous biomass into various bioassimilable products. Therefore, it has taken center stage in numerous biotechnological industries. These unique inducible enzyme cocktails are well known for their distinctive ability to reduce disulfide bonds in keratin, followed by peptide bond hydrolysis, demonstrating potential to replace current conventional methods of treating keratinous waste. The three microbial domains reported to have expressed keratinolytic activity include bacteria [21,22,23], fungi [24,25,26], and actinomycetes [27,28]. The opportunities presented by keratinases may lead to the generation of cost-effective and environmentally friendly products while retaining excellent integrity due to sustainable valorization process conditions. Even though keratinolytic enzyme cocktails demonstrate robust catalytic tendencies, they recently gained momentum in industrial biotechnology for accelerating waste bioresource recycling and recovery. Hence, most studies are on isolating keratinolytic enzymes and their application in disintegrating tough and insoluble keratinous biomass into high-value products with multifaceted application potential. Thus, a few review articles previously published examined the application prospects of microbial keratinase, considering the information readily available then [4,29,30,31]. Therefore, the continuous evolution of research findings highlights the need to piece together the updated information on these enzymes to shed light on their economic importance and possible industrial applications. Therefore, this review discussed the economic significance of keratinous waste in the environment. It identified different microbial sources of keratinolytic enzymes while examining the sustainable conversion of keratinous biomass using enzyme and microbial technology. The study presented the optimization strategies for improving keratinases’ output and discussed various avenues for their prospective application. It also surveyed the techno-economic considerations for industrial-scale keratinase applications and highlighted the current limitations and existing knowledge gaps for future research direction.

## 2. Keratinous Waste Biomass in the Environment

The need to process chicken, fish, birds, and reptiles for food is pertinent for sustaining the burgeoning global population. However, the waste materials associated with these products are typically disposed of with little or no recycling, thus posing adverse effects on human health and the environment. Methods of disposing of keratinous wastes include incineration, composting, open-air burning, and landfilling, causing ecological perturbation and increasing the carbon footprint [32]. The landfilling of this stubborn biomass is associated with foul smell, flies and rodents’ infestation, water and soil pollution [3]. The leather industry significantly contributes to air, water, and soil pollution, producing large amounts of solid and liquid keratin wastes of animal origin [33]. Additionally, keratinous wastes are hotspots for pathogenic bacteria, especially chicken feathers, while human hair contributes to clogging the drainage systems [34,35,36,37].

**Figure 1:**
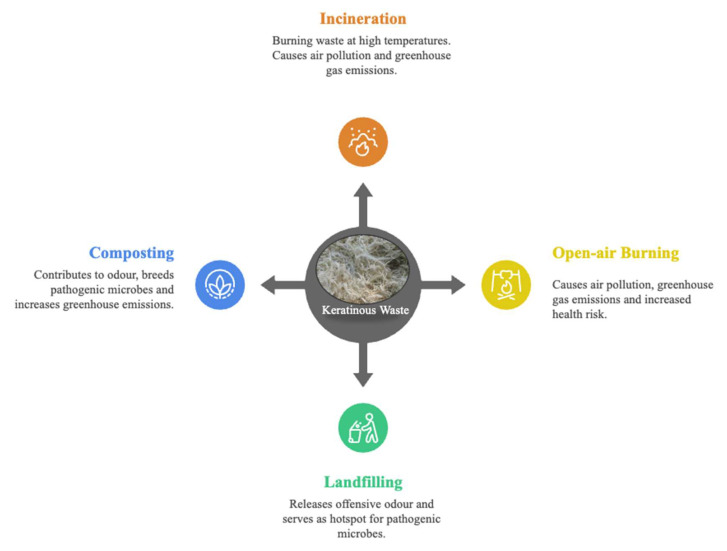
A schematic representation of different traditional methods of keratinous waste disposal and their respective negative impacts on the environment.

The nitrogen composition of the keratinous biomass is approximately 15%; thus, composting has become one of the best options for recycling and disposing of this biomass to reduce its environmental littering. However, similar to other disposal methods, composting indirectly affects the ecosystem, as it causes water pollution and releases greenhouse gases, thus contributing to the greenhouse effect [38,39,40] (Figure 1). These negative impacts on the ecological landscape associated with the traditional methods of biomass handling highlight the need for alternative and eco-friendly methods that promote a circular economy. Thus, keratinolytic enzymes efficiently convert keratinous biomass into functional peptides.

## 3. Recycling Keratinous Biomass Using Sustainable Technology

The biological approach of using keratinases to convert keratin waste into soluble proteins and amino acids represents a sustainable technology that has proven to enhance the economic value of keratinous wastes. Unlike the chemical extraction method of keratin, which requires a reducing sulfite reagent to uncouple the cross-linked disulfide bonds, keratinolytic microflora secrete disulfide reductases. These enzymes initiate keratin hydrolysis and are continuously secreted during the process. Three steps have been proposed to cause keratin hydrolysis: sulfitolysis, proteolysis and peptidolysis [41]. Sulfitolysis is the first step, where keratinases bind to keratinous substrates and disrupt the tightly packed disulfide bonds, altering the keratin configuration and exposing multiple hydrolytic sites for keratinolytic proteases. The accumulation of oligopeptides in the medium initiates further peptidolytic attack, generating small peptides and amino acids.

**Figure 2:**
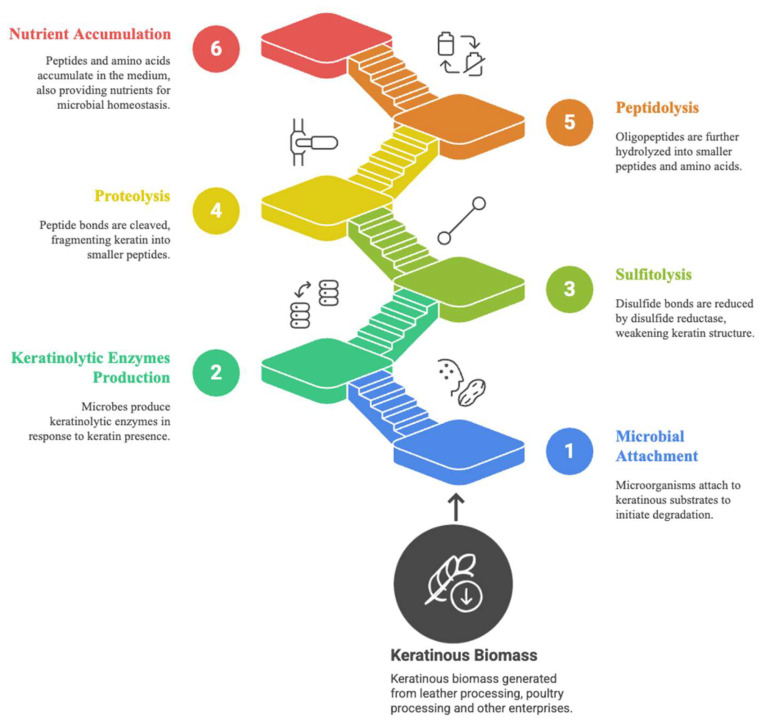
The stepwise processes involved in keratinous biomass degradation by keratinolytic strains. Attachment of the microorganism to the biomass elicits the expression of keratinolytic enzyme system. Sulfitolysis entails reduction in densely populated disulfide bonds. Proteolysis involves the cleavage of peptide bonds exposed by sulfitolysis. Peptidolysis implies further digestion of the oligopeptides to generate hydrolysates rich in amino acids and short peptides.

Keratinolytic microflora completely degrade keratinous biomass in four sequential steps: adhesion, colonization, production of keratinolytic enzymes, and breakdown of the keratin substrate, as illustrated in Figure 2 [2,42]. The first step, adhesion, occurs when keratinolytic microflora bind to the keratin. The microbial organisms then colonize the surface of the keratinous biomass and begin expressing the relevant ancillary enzymes for degrading biomass components other than keratin. The removal of these components exposes the keratin macromolecular structure to enzymatic attacks [43]. The secreted keratinolytic enzyme cocktails then reduce the closely packed disulfide bonds in the keratin polymer. This disulfide bond reduction encourages subsequent digestion by exo- and endo-oligopeptidases, generating free amino acids and short peptides.

## 4. Sources of Microbial Keratinases

Keratinolytic activity has been reported in three domains of life: actinomycetes, fungi, and bacteria [44,45,46,47,48,49]. Their diverse physiological preferences and metabolic capabilities have made these microbial strains ubiquitous in soil and environments with high keratinous biomass, such as slaughterhouses, the leather industry, textile facilities, and poultry processing plants [50,51].

### 4.1. Bacteria as Sources of Keratin-Degrading Enzymes

Bacteria are one of the diverse microbial species that have demonstrated the ability to produce keratinases when subjected to a keratinous substrate. Among the identified keratinolytic bacteria, Gram-positives are the most keratinase-producing bacteria, especially the *Bacillus* spp. [1,52,53]. The *Bacillus* genus that has been observed to possess the ability to secrete keratinases includes *B. subtilis*, *B. pumilus*, *B. licheniformis*, and *B. cereus.* Keratinases from *B. licheniformis* have been exhaustively characterized and are already in commercial use [54,55,56]. Other Gram-positive bacteria with keratinolytic activity include *Microbacterium* spp., *Lysobacter* spp., *Nesternokia* spp., and *Kocuria* spp. [1,51]. *Streptomyces* spp. including *Streptomyces griseoaurantiacus*, *Streptomyces pactum*, *Streptomyces thermoviolaceus*, and *Streptomyces albidoflavus* have been implicated with keratinolytic potential [57,58,59]. However, some Gram-negative bacteria, such as *Vibrio* spp., *Chryseobacterium* spp., *Xanthomonas* spp., *Burkholderia* spp., *Pseudomonas* spp., *Thermoanaerobacter* spp., and *Fervidobacterium* spp., have also demonstrated excellent keratinolytic activity [1,53,60,61].

A litany of different bacterial species with keratinolytic potential is isolated from diverse environments, which include dump sites, poultry processing farms, soil, decomposing organic matter, and slaughterhouses. Soil is an abundant source of keratinolytic bacteria, and some potent strains, including *Streptomyces thermoviolaceus* [62], *Bacillus subtilis* S14 [63], and *B. licheniformis* ER-15 [64] have been isolated and characterized. The poultry processing farms have also proven to be hotspots for isolating bacteria with keratin-degrading dexterity because of their constant exposure to this biomass. Table 1 shows selected keratinolytic bacteria with their respective isolation sources.

### 4.2. Fungi as Sources of Keratin-Degrading Enzymes

The fungal kingdom is one of the domains of life that has proven to be an excellent producer of keratinases since 1952, after the discovery of the hair-baiting technique [78]. Keratinolytic fungi have been identified to belong to different genera, including *Fusarium*, *Myrothecium*, *Aspergillus*, *Doratomyces*, *Scopulariopsis*, *Microsporum*, *Trichoderma*, *Purpureocillium*, and *Penicillium* [37,49,79]. These keratinolytic fungal species are mostly copious in soil environments, possessing a wide range of temperatures (15 to 35 °C). They play a huge role in the biodegradation of keratinous waste and other agricultural biomass, contributing to the nutrient recycling in the soil.

Although many fungal species are known for their excellent keratinolytic activity, a few reports have been published on fungal keratinases. Several reports have mainly focused on bacterial keratinases [1,20,21,58,80,81,82]. In the kingdom Fungi, two groups are best known for their keratinolytic activity: the Deuteromycetes and the Ascomycetes. Fungal species with keratinolytic activity are abundant in various habitats, including soils (mainly agricultural soils), extreme cold environments such as Antarctica, and extremely hot and humid environments [83].

The keratinases from fungi have displayed extraordinary biodegradation of keratinous biomass; however, most of these keratinolytic fungal species are also known for their pathogenicity [84]. Interestingly, a few keratinolytic fungi have been fully characterized, including *D. microspores*, *P. marquandii* [85], *O. corvina* [86], *T. album* [87], *A. parasiticus* [88], and *M. verrucaria* [89]. Fungal keratinases present an attractive approach for degrading keratinous materials into functional peptides, thereby contributing to the eco-friendly recycling of waste biomass for a sustainable circular economy.

### 4.3. Actinomycetes as Sources of Keratin-Degrading Enzymes

The actinomycetes are a large group of Gram-positive bacteria known for their resemblance to fungi in some morphological traits, such as filamentous growth and sporulation, which differentiate them from other bacterial strains [58]. This group of bacteria holds immense roles in numerous commercially important applications, which include biodegradation of contaminants and bioactive compounds production, including antibiotics and enzymes [90,91]. Among the special traits possessed by the Actinomycetes, the production of industrially important enzymes, such as lipases, amylases, cellulases, xylanases, proteases and chitinases, among others, are predominantly high. Among the commercially important enzymes produced by the Actinomycetes, keratinases have been identified, confirming their participation in keratin degradation.

Among the characterized keratinolytic actinomycetes, *Streptomyces* is the leading keratinase producer [92]. The reported keratinolytic *Streptomyces* include *S. griseus* [93], *S. fradiae* [94], *S. pactum* [95], *S. thermoviolaceus* SD8 [62], and *S. graminofaciens* [96]. The other identified keratinolytic Actinomycetes include *T. candidus* [52,53], *Actinomadura* [97], *Kocuria* [98], *Kytococcus* [99], *Microbacterium* [40,41], *Thermoactinomyces* [100], and *Nocardiopsis* [101].

The keratinolytic actinomycetes group are exceptionally abundant in diverse environments, which include marine environs [102], poultry waste [97], keratinous waste dumpsites [91], rice mill wasteyard [103], and soil [104,105,106]. However, as highly talented as the Actinomycetes are in producing biocatalytic enzymes, few reports have been published on their keratinase production ability. Therefore, researchers must focus on exploring diverse species of actinomycete strains for potential novel keratinolytic enzymes with commercial prospects.

### 4.4. Comparative Analysis of Keratinase Production Among the Keratinolytic Microbial Strains

Microbial keratinases present a great diversity in their biochemical and biophysical properties, including pH, temperature, and molecular weight, and they are majorly serine-metallo proteases (Table 2). Keratinolytic strains of the same genus can secrete keratinases with dissimilar biochemical properties. Additionally, amino acid sequences of keratinolytic enzymes from fungi and actinomyces differ from those produced by bacteria. The three domains, fungi, bacteria, and Actinomycetes, have demonstrated their ability to completely degrade keratinous-based biomass through the extracellular secretion of keratinolytic proteases; however, bacteria are the predominant keratin degraders, particularly the *Bacillus* spp. [1,52,53]. Keratinolytic bacteria have gained momentum industrially because of their keratinolytic activity, short generation time, and ease of maneuverability.

Also, bacteria thrive under extreme and fluctuating environmental conditions, and their enzymes remain catalytically active [6]. Bacterial keratinases have been reported to possess an optimal temperature range of 28–90 °C, and a pH range of 5.0–11 [12]; however, some extremophiles have been likewise documented. Most of the fungal keratinases are identified from dermophytes, limiting their commercial prospects, thus promoting bacterial keratinase as the candidate for industrial applications [6]. The majority of industrial products developed with keratinolytic protease are from *Bacillus* sp. keratinases, including Versazyme™, the first thermo-resistant commercial keratinase from *Bacillus licheniformis* PWD-1 developed by Shih and coworkers at Bioresource International, Inc., Durham, NC, USA.

**Table 2:** Different keratinolytic strains and the biochemical properties of the keratinases they secrete.

Keratinolytic Strain	Domain	Enzyme Type	Optimum pH	Optimum Temperature (°C)	Molecular Weight (kDa)	Reference
*Chryseobacterium* sp. Kr6	Bacteria	Metallo	8.5	50	64	[107]
*Clostridium sporogenes*	Bacteria	-	8	55	28.7	[108]
*Bacillus licheniformis* PWD-1	Bacteria	Serine	7.5	50	33	[109]
*Bacillus cereus* DCUW	Bacteria	Serine	8.5	50	50	[110]
*Bacillus licheniformis* FK14	Bacteria	Serine	8.5	60	35	[111]
*Bacillus licheniformis* K-508	Bacteria	Thiol	8.5	52	42	[112]
*Bacillus licheniformis* RPk	Bacteria	Serine	9	60	32	[113]
*Bacillus subtilis* MTCC (9102)	Bacteria	Metallo	6	49	69	[114]
*Streptomyces albidoflavus*	Actinomycetes	Serine	6.0–9.5	40–70	18	[68]
*Streptomyces pactum*	Actinomycetes	Serine	7–10	40–75	30	[95]
*Streptomyces thermoviolaceus*	Actinomycetes	-	8	55	40	[98]
*Aspergillus fumigatus*	Fungi	Serine	6.5–9	45	-	[115]
*Aspergillus oryzae*	Fungi	Metallo	8	50	60	[116]
*Myrothecium verrucaria*	Fungi	Serine	8.3	37	22	[89]
*Paecilomyces marquandii*	Fungi	Serine	8	60–65	33	[85]
*Scopulariopsis brevicaulis*	Fungi	Serine	8	40	36–39	[117]
*Trichophyton schoenleinii*	Fungi	-	5.5	50	38	[118]
*Trichophyton vanbreuseghemii*	Fungi	Serine	8	-	37	[119]

## 5. Isolation of Keratinolytic Microorganisms and Production of Keratinases for Prospective Applications

The isolation technique, mostly effective for recovering potent keratinolytic strains, involves constructing an enrichment medium. Enzyme production by keratinolytic microbial strains is achieved with either solid-state fermentation or submerged fermentation. Firstly, a sample source is identified, which includes soil, chicken feathers, poultry waste, soil from slaughterhouses, contaminated water with keratinous biomass, and animal keratinous tissues, such as horns, hooves, hair, and nails [106,120,121,122]. The samples are inoculated in the medium of a keratinous substrate as the only carbon and nitrogen source. The keratinous substrate varies, with numerous options, including chicken feathers, human hair, wool, and bovine hair. Chicken feathers are the most widely used keratinous substrate for developing an enrichment medium [53,123]. As the sole carbon and nitrogen source, the presence of keratinous substrate induces the expression of keratinase for subsequent biomass degradation into bioaccessible products [124]. Microbes utilize these soluble products in the medium for nutrient and energy requirements. The degradation of the supplemented keratinous substrate phenotypically denotes the organism’s keratinolytic activity. The degradation ability varies depending on microbial metabolic capabilities [52].

The isolation method could focus on single-strain recovery or microbial consortia development, depending on the research interest. Microbial consortia represent the great strategy of managing keratinous waste on an industrial scale due to the metabolic cooperation of different microbial strains [125,126]. Visual inspection does not validate the keratinolytic potential of the strain vis-à-vis the degree of keratin hydrolysis and extracellular keratinase production. Therefore, measuring the keratinolytic activity via enzyme assay and quantifying residual keratin biomass are used to evaluate the keratinolytic prospects of each microbial strain [127]. Furthermore, methods including microbiological and biochemical techniques, polymerase chain reaction, 16S rRNA gene sequencing, next-generation whole genome sequencing and phylogenetic analysis are used to confirm the identities of keratinolytic microbial species. Interestingly, the whole genome remains the best identification technique, as knowing the genome of the keratinolytic strain provides insight into understanding the keratinolytic mechanism and pathways [128].

Although microbial keratinases represent an excellent endeavour in the bioconversion of keratinous waste, their production by microbial species does not yet meet industrial scale. Consequently, microbial keratinase production is optimized by adjusting the physicochemical parameters. These parameters include substrate concentration, inhibitors, surfactants, chemical solvents, metal ions, incubation temperature and medium pH.

### 5.1. Optimizing pH Conditions for Efficient Keratinase Production

The pH of the fermentation media plays a vital role in inducing keratinase production. The best physicochemical variables associated with keratinase production have been determined using either a classical approach or statistical method, and it has been observed that most keratinases are produced under neutral to slightly alkaline conditions, with the optimum pH being between 7 and 9 [129,130,131,132,133]. Even though keratinase production optimally occurs at neutral and slightly acidic conditions, some microbial strains are highly alkalophilic. For example, *Bacillus thuringiensis* TS2 maximally expressed extracellular keratinase at an optimum pH of 10, and *Penicillium* species displayed keratinolytic activity at a pH optimum of 10–11 [134,135].

Other examples of extreme alkalophilic keratinolytic strains include *Nocardiopsis* sp. strain TOA-1 [136], *Streptomyces* sp. AB1 [106], and *Bacillus circulans* [137]. The neutral to slightly alkaline pH optima have been suggested to encourage transport of the produced keratinase from the inside microbial compartments to the extracellular medium [120]. As much as keratinolytic microbes portrayed their enzyme production efficacy at an alkaline range of the spectrum, a few keratinolytic strains producing keratinases at acidic pH have been identified. These include *S. pactum* DSM40530 [95], which had keratinolytic activity at pH 4, *Nocardiopsis* TOA-1 with keratinolytic activity at pH 1.5 to 12 [101], and *Candida* species with pH optima of 5.5 to 9 [138]. According to Gupta and Ramnani [30], keratinolytic enzymes generally maintained biocatalytic functionality over a broad pH range of 5 to 13, affirming their wide production pH range.

### 5.2. Optimizing Temperature for Efficient Keratinase Production

Temperature is another significant factor driving keratinase production. These enzymes exhibit excellent activity at a wide range of temperatures depending on the microbial source. Adjusting the temperature during fermentation influences the keratinase production. The keratinases have been observed to be best produced at temperatures ranging from 30 °C to 80 °C [30]. Commonly, the bacterial keratinase production optimally occurs from 25 to 50 °C, while actinomycetes and fungi can vary significantly [60,139]. Keratinase production at a high temperature has been suggested to support excellent keratin degradation by keratinolytic microbes [140]. Disulfide bonds primarily orchestrate the stability in keratinous material [141]. However, some keratinolytic strains express keratinases at extreme temperatures; for example, *Chrysosporium keratinophilum* and thermophile *Fervidobacterium islandicum* AW-1 demonstrated keratinolytic activity at higher temperature conditions [43,60].

### 5.3. Optimizing Keratinous Substrate for Enhanced Keratinase Production

The substrate preference depends on the microbial keratinase producer. Keratin substrates used in keratinase production can be either natural keratin or substrates derived from keratin-rich materials. Natural keratin substrates include feathers, pig bristles, cow horn, hair, and wool, while keratin-derived substrates include keratin powder and azo keratin [5,30,142]. Even though keratinases show activity against a broad spectrum of substrates, feathers have been observed to be the substrate associated with high keratinolytic activity. For instance, *Scopulariopsis brevicaulis* expressed high keratinolytic activity (82.53 U mL^−1^) on chicken feathers, and the activity dropped to 41.20 U mL^−1^ on human nails as a substrate and lower to 36.17 U mL^−1^ on human hair [143].

Likewise, mutant *Bacillus subtilis* KD-N2 was subjected to different substrates (i.e., feathers, wool, hair, and silk) for keratinolytic activity, and the strain demonstrated optimum activity on the feather substrate [48]. Similarly, Riffel et al. [107] reported *Chryseobacterium* sp. kr6 with optimum keratinolytic activity on a feather substrate. Additionally, Moridshahi et al. [144] reported *Bacillus* sp. BK111 with chicken feather substrate as a preferred keratinous substrate. However, other strains display unmatched keratinolytic activity on other keratinous substrates compared to those in the feather-formulated medium. For instance, *Arthrobacter* sp. NFH5 demonstrated unsurpassed activity in keratin powder compared to feathers as substrates [71]. *Aspergillus oryzae* exhibited remarkable activity on soluble bovine serum albumin and casein compared to the feather substrate [116]. This observation highlights that the nature of the keratinous substrate hugely influences keratinase production. Therefore, selecting the best keratinous substrate to enhance keratinase production when developing the fermentation media is important.

### 5.4. Optimizing Metal Ions for Enhanced Keratinase Production

Undoubtedly, microbial keratinases have displayed an impactful potential in managing and recycling keratinous waste and have therefore taken a prominent role in industry and biotechnology. The immense potential of keratinolytic enzymes in industrial biotechnology has resulted in extensive research on enhancing the production and activity of these enzymes to meet industrial scale. Some metal ions, which include Mg^2+^, Ba^2+^, Ca^2+^, Fe^2+^, Mn^2+^, K^+^, Co^2+^, and Li^+^ have been discovered to significantly enhance keratinase production [60,95,145]. The presence of these metal ions in the cultivation media stimulates the enzyme’s secretion and supports their catalytic efficiency [137,146,147]. The enhanced keratinolytic activity in the presence of these metals is suspected to be associated with the protection of the enzyme against denaturation by metal ions; hence, these ions are stabilizing agents or keratinase activators [11,60,68].

## 6. Transformation of Keratinous Waste Through Microbial Keratinolysis: Application Prospects

**Figure 3:**
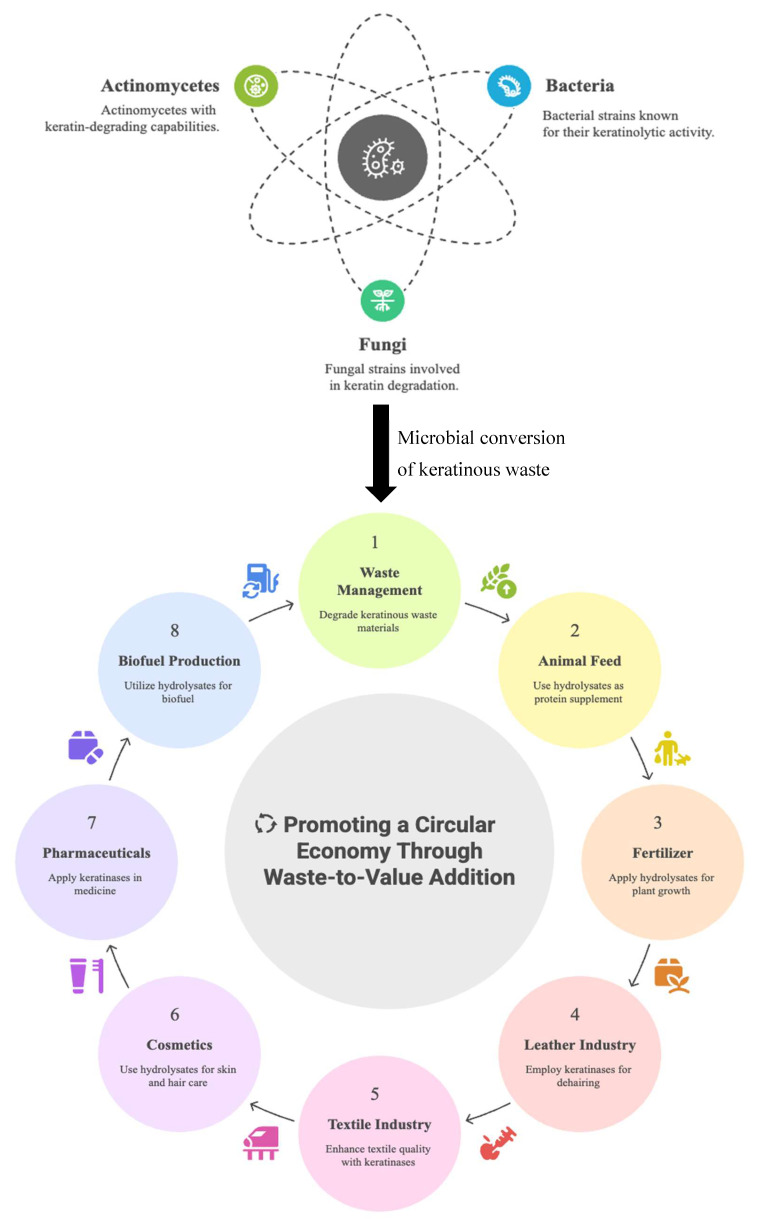
Schematic display of a sustainable management of keratinous waste and prospective applications of keratinases and hydrolysates produced during microbial conversion of keratinous biomass.

The potential applications of microbial keratinolysis have lured researchers to explore keratinase-aided valorization of keratinous biomass and maximize the keratinase outputs. Keratinolytic microbes and the associated keratinases have presented a wide range of biotechnological and industrial applications that include but are not limited to animal feedstock production, waste management, pharmaceutical and biomedical, leather and bioenergy, detergent formulation and agriculture (Figure 3) [8,50,135,148].

### 6.1. Keratinases in Waste Management and Recycling

Microbial keratinases have increasingly gained centre stage in waste recycling for several types of keratinous biomass, including poultry waste, slaughterhouse waste, leather and textile processing byproducts. For over two decades, keratinous waste from various enterprises has been discarded indiscriminately or poorly recycled [31]. The teeming global population has fanned the flames in the production of keratinous waste by pressuring the agro-industry to upscale the processing of livestock products. To date, slaughterhouses, poultry processing, leather, wool and textile processing are accountable for generating more than 8.5 million tons of keratinous wastes [42,50,149,150]. Consequently, these massive keratinous wastes harm the environment; for example, chicken feathers are massively produced from poultry processing without proper handling. They persist in the environment due to the keratin content, constituting various pollution problems, including releasing offensive smells and breeding pathogenic microbes [12,151].

Due to the regulatory policies prohibiting indiscriminate dumping of waste in the environment, microbial conversion of keratinous waste into value-added products represents a sustainable strategy from economic and ecological perspectives [100,152,153]. Therefore, there is a need for further development of this research field to upscale the waste-to-wealth generation. The advancement of research in developing robust microbial strains for efficient keratinous waste recycling augurs well for industrial-scale biomanufacturing.

### 6.2. Keratinases in the Agricultural Sector

The agricultural sector is one of the potential beneficiaries of the opportunity presented by microbial keratinases. Agricultural wastes such as chicken feathers contain a high amount of crude protein in keratin, making them rich in diverse amino acids, including serine, glutamic acid, proline, and small amounts of methionine, histidine, and lysine [50]. The microbial conversion of keratinous biomass results in feedstocks with multifaceted applicability for a circular economy [154,155].

Poultry waste, such as chicken feathers, has since been used in animal feed. However, the challenges of compromised nutrient quality due to the preparation method have limited its commercialization. Hence, the microbial keratinases-mediated degradation of keratinous biomass represents an eco-smart bio-recycling of chicken feathers, enhancing the nutrient bioavailability for livestock production. Bacterial strains, such as *Vibrio* spp. and *Streptomyces* spp. keratinolytic proteases effectively hydrolyzed keratinous biomass to produce a high-nutritional feed supplement [16,156]. On the other hand, the *Bacillus* spp., including *B. pumilus* A1, *B. licheniformis* PWD-1, *B. licheniformis* LMUB05, and *B. licheniformis* ER-15, have demonstrated their ability to degrade chicken feathers into hydrolysates with significant amino acid composition [157,158,159]. These keratinase-derived protein supplements were reported to improve the animal immune system, promote growth and provide an alternative, cheap feed for animal husbandry [155].

The agricultural sector is adopting an eco-friendly manure for soil fertility amendment due to the ecological decay posed by inorganic fertilizers. Organic fertilizers are typically used to encourage crop production, ensuring the food supply is enough for the growing population. Ideally, fertilizer is rich in phosphate, nitrogen, and carbon, and keratinous waste biomass holds an immense amount of these organic compounds. Therefore, keratinous biomass is an excellent source for organic fertilizer preparations [160]. Bio-fertilizers from chicken feather digestion not only improve the crop’s quality but also improve the soil quality. The keratinase-based bio-fertilizers promote plant growth, phosphate solubility, water retention capacity, and microbial activities [8,122,146].

An increased energy index, plant growth, and feed conversion ratio of 30% in rice were observed in rice seeds planted on soil amended with keratinase-treated feathers from *Bacillus* spp. AJ4 and AJ9 [161]. Bovine hooves and horns treated with keratinases from *Paecilomyces marquandii* are an excellent bio-fertilizer for improving plant growth [162]. Furthermore, bio-fertilizer prepared from feathers treated with keratinases from *Paenibacillus woosongensis* TKB2 improved nodule formation, soil fertility, and germination of seeds in *Cicer arietinum* [163]. Therefore, keratinase-based organic fertilizers are rich in phytohormones and plant nutrients for promoting plant growth and performance, soil quality, crop yield, cost-effectiveness and eco-friendliness.

### 6.3. Keratinases in Cosmetics Production

Keratin hydrolysates have been included in the formulation of numerous hair and skin products, including hair shampoo, bath soaps, acne treatments, and skin moisturizers. In the skin, pure keratin provides moisture, thereby preserving the skin’s natural integrity, while in hair, it enhances the mechanical and thermal properties of the hair [164,165]. The keratinase-mediated keratin hydrolysates act as a preservation and moisturizing agent that binds water from the lower layers of the epidermis to the stratum corneum and hair cuticle. Moreover, keratinase-produced keratins are excellent formulations in whitening, bleaching, and acne treatment cosmetics [166]. Keratinase-based hair moisturizers and hair conditioners are excellent in preventing and repairing damaged hair [167]. While in skin products, keratinase-based cosmetics help repair dead skin tissues and unclog sebum-forming cells [168].

### 6.4. Keratinases in Detergent Formulation

Recently, enzyme-based detergents have been the preferred choice over chemical-based detergents, as they possess exceptional cleaning features, such as fabric fiber compatibility, biodegradability, stubborn dirt-removing properties, and low-temperature washing tolerance. Keratinases are thermostable, wide pH and surfactant-tolerant, securing a top spot in the detergent industry as bio-additive [70]. The broad substrate specificity of keratinases has promoted their detergent performance [169].

The stain removal ability of keratinase was tested using extremely filthy collars and cuffs, and it was observed that the detergents combined with keratinase exceptionally cleaned the collars and cuffs without damaging the cotton’s texture [137]. After treatment, a keratinase from *P. woosongensis* TKB2 removed tough blood, egg yolk, and chocolate stains [163]. On the other hand, Sivakumar et al. [147] reported impressive cleaning efficiency of blood and egg stains by a keratinase sourced from *B. thuringiensis* TS2. Keratinases could also degrade keratinous waste blocking drainages, such as hair, feathers, and scales, highlighting the potential of microbial keratinases in sustainable development.

### 6.5. Keratinases in Leather and Textile Production

Leather and textile production provide basic needs and contribute towards economic growth and development. Nevertheless, the toxic chemicals released while processing leather and textiles pose serious health challenges to humankind [170]. For instance, certain harmful substances such as sodium sulphate are used for pre-tanning and dehairing animal skin. Sodium sulphate constitutes environmental challenges, affecting the chemical oxygen demand, biological oxygen demand, and total suspended solids in the effluent samples and receiving water bodies [8,9,135,171]. The replacement of chemical agents with biocatalysts in processing leather materials offers a more sustainable and ecologically friendly approach.

Microbial keratinases have demonstrated the capacity to competently dehair animal hides, improve leather quality, be eco-friendly, and decrease environmental pollution [172]. Keratinases with excellent properties for leather treatment have been isolated in various microbial species, including *B. subtilis* KD-N2, *Trichoderma harzianum* MH20, *B. subtilis* S14, and *P. woosongensis* TKB2 [14,163]. Other keratinolytic strains that have showcased their importance in leather processing include *Aspergillus nidulans* and *Bacillus* sp. PPKS-2 [35,74,137,173].

Wool production is another important growing process in the textile industry but has several challenges. Wool is rich in keratin; thus, classical wool processing requires chemical treatment due to its resistance to degradation. Absorbable organic chlorides are used for processing wool; however, these treatments alter the texture of the wool, are energy-consuming, and generate highly toxic wastewater [150]. Using keratinolytic proteases, wool can be processed without losing the desired strength and texture [151,174]. On the other hand, keratinases are biodegradable; hence, they can tackle the environmental challenges associated with wool production.

### 6.6. Keratinases in Medicine and Pharmaceuticals

Keratinases have gained popularity in the pharmaceutical sector and medicine due to their potential efficacy in treating stubborn skin conditions like calluses, psoriasis, and acne. Keratinases liquefy dead skin in acne, blocking the sebaceous gland [150]. Keratinases have also proved to replace chemical agents such as glycerin, dichlorobenzene, hydrogen peroxide, sodium bicarbonate, carbamide peroxide, and triethanolamine for cerumen treatment [175]. Study has also underscored their potential role in prion decontamination, broadening their biomedical importance [150].

## 7. Keratinolytic Enzyme-Keratinous Waste-Product Relationships

Effective biodegradation of keratinous waste results in the liberation of variable products in the keratin hydrolysates. Analysis of the hydrolysates shows that keratinolytic enzymes dismember keratin into amino acids, dipeptides, oligopeptides, ammonia, and other soluble proteins [176,177]. It is an established concept that cooperative actions of disulfide reductases and keratinases facilitate keratin bio-digestion into its composite units. Keratinase-mediated complete hydrolysis of keratinous biomass, regardless of the source, generally involves catalytic attack by endopeptidases, exopeptidases and oligopeptidases [49,178]. After disulfide bond reduction, endopeptidases recognize and cleave keratin from the inside of the polypeptide, while exopeptidases cut the polymer from the extremes. Oligopeptidases further digest the oligopeptides emanating from the actions of the two peptidases already mentioned, yielding short peptides and amino acids. Table 3 summarily shows the sustainable bioconversion of keratinous biomass into protein hydrolysates, degradation products’ identification strategies and application potential. Researchers seldom separate these crucial steps of keratin degradation since they happen simultaneously in the presence of keratinolytic enzyme cocktails, and the whole processes are always credited to keratinase action.

Consequently, keratinase KerZ1 from *Bacillus subtilis* digested keratinous feathers, releasing soluble hydrolysates with relatively high abundance of glutamic acid, alanine, tyrosine, phenylalanine, leucine and lysine [179]. The protein hydrolysate also showed the presence of mixed short peptides with a molecular weight around 1.3 kDa; applying mass spectrometry further identified up to 12 bioactive peptides. Keratinolytic *Stenotrophomonas maltophilia* BB11-1 was used to degrade native wool in a 30 L fermenter, and the wool hydrolysate profiling revealed that wool keratin predominantly consisted of alpha helical protein chain as observed by the appearance of amide I (–CO–NH–) peak at 1650 cm^−1^ [180]. An additional amide II (–N–H–) absorption peak close to 1519 cm^−1^ suggests the presence of peptides resulting from wool hydrolysis by strain BB11-1 Keratinolytic enzyme. Amino acid analysis using an amino acid analyzer showed that the wool hydrolysate contained about 17 amino acids with high concentrations of phenylalanine, aspartic acid, glutamic acid, and cysteine [180]. Kshetri and colleagues characterized peptides from chicken feather hydrolysates fermented with locally isolated *Streptomyces tanashiensis* RCM-SSR-6 and *Bacillus* sp. RCM-SSR-102 [181]. Fourier Transform Infrared Spectroscopy examination of the hydrolysates demonstrated that the peptide constituents of the hydrolysates displayed amide I and amide II peaks in the spectral range of 1700–1600 cm^−1^ and 1600–1500 cm^−1^, respectively. Keratin hydrolysates obtained by degrading feathers with keratinase-producing *Bacillus subtilis* AMR showed high concentration of peptides (800 to 1079 Da) after analysis using Matrix-Assisted Laser Desorption/Ionization Time-of-Flight (MALDI-TOF) mass spectrometry [167]. Further investigation using High Performance Thin-Layer Chromatography showed the presence of lower molecular mass peptides and amino acids in the protein hydrolysates. Wan et al. [182] characterized a novel peptide with antioxidant activity from feather hydrolysates generated by *Bacillus subtilis* S1-4. Analysis of the hydrolysate using MALDI-TOF elucidated the peptide identity with the amino acid sequence of Ser-Asn-Leu-Cys-Arg-Pro-Cys-Gly [182]. A harsh environmental condition or biological activity of other metabolites facilitates the deamination of amino acids and other soluble proteins in the keratin hydrolysates, accumulating ammonia in the cultivation medium [176].

**Table 3:** Summary of keratinous biomass bioconversion, degradation products identification and potential uses.

Source of Keratin Hydrolysate	Biotreatment Agent	Product Identification Method	Identified Products	Potential Uses	References
Animal hair	*Brevibacterium luteolum* MTCC 5982	HPLC	Amino acids (Asp, Glu, Cys, Ser, His, Gly, Thr, Arg, Ala, Tyr, Met, Val, Phe, Ile, Leu, Lys)	-	[171]
Chicken feathers	ICSE coupled with keratinase	HPLC-MS/MS Amino acid auto-analyzer	Peptides (500 Da, <3 kDa) Amino acids (Asp, Thr, Ser, Glu, Gly, Ala, Cys, Val, Met, Ile, Leu, Tyr, Phe, His, Lys, Arg, Pro)	Antimicrobial	[183]
Feathers	*Bacillus subtilis* S1-4	RP-FPLC, MALDI-TOF/TOF-MS/MS	Peptide (Sequence: Ser-Asn-Leu-Cys-Arg-Pro-Cys-Gly)	Antioxidant	[182]
Chicken feathers	*Bacillus velezensis* NCIM 5802	NMR, ESI-MS	Amino acids (Thr, Pro, Val, Asn, Leu, Ile, Ser, Asp, Glu, Gln, Lys, Arg, His, Phe, Tyr, Met, Cys, Trp)	-	[184]
Sheep wool	Recombinant *Bacillus subtilis*	Amino acid analyzer	Amino acids (Asp, Thr, Ser, Glu, Pro, Gly, Ala, Cys, Val, Met, Ile, Leu, Tyr, Phe, His, Lys, Arg)	-	[185]
Chicken feathers	*Chryseobacterium sediminis* RCM-SSR-7	HPLC	Amino acids (Asp, Glu, Ser, His, Gly, Thr, Arg, Ala, Tyr, Met, Val, Phe, Ile, Leu, Lys)	Feed supplement, Organic fertilizer	[186]
Chicken feathers	Recombinant *Bacillus subtilis* WB600	Amino acid analyzer	Amino acids (Asp, Thr, Ser, Glu, Gly, Ala, Cys, Val, Met, Ile, Leu, Tyr, Phe, His, Lys, Arg, Pro)	-	[187]
Chicken feathers	Keratinolytic bacteria, keratinase	FTIR	Peptides (<10 kDa)	Antioxidant, Antityrosinase	[181]
Chicken feathers	Ketatinolytic enzyme	-	Peptides (<3 kDa)	Antioxidant	[188]
Chicken feathers	Keratinolytic *Rhodococcus erythropolis*	RP-HPLC, MALDI-TOF, FTIR	Peptides (3861 Da)	Antibacterial, Antibiofilm	[189]
Chicken feathers	*Chryseobacterium* sp. kr6	LC-MS/MS	Peptides (1155.641 Da)	Antioxidant	[190]
Feathers	Keratinase	UPLC/Q-TOF-MS	Peptides	ACE inhibitor, DPP IV inhibitor	[191]
Chicken feathers	*Bacillus licheniformis* WHU, Keratinase	LC-MS	Peptides Amino acids (Trp, Tyr, Asp, Thr, Ser, Glu, Ala, Val, Met, Ile, Leu, Phe, His, Lys, Arg, Pro)	Antioxidant Feed supplement	[192]

HPLC-MS/MS—High Performance Liquid Chromatography with tandem Mass Spectrometry; ICSE—Instant catapult steam explosion; Asp—Aspartate; Thr—Threonine; Ser—Serine; Glu—Glutamate; Gly—Glycine; Ala—Alanine; Cys—Cysteine; Val—Valine; Met—Methionine; Ile—Isoleucine; Leu—Leucine; Tyr—Tyrosine; Phe—Phenylalanine; His—Histidine; Lys—Lysine; Arg—Arginine; Pro—Proline; Asn—Asparagine; Gln—Glutamine; FTIR—Fourier Transform Infrared Spectroscopy; RP—Reverse phase; LC-MS/MS—Liquid Chromatography and tandem Mass spectroscopy; MALDI-TOF—Matrix-Assisted Laser Desorption/Ionization Time-of-Flight; UPLC/Q—Ultra-Performance Liquid Chromatography/Quadrupole; ESI-MS—Electrospray Ionization Mass Spectrometry; NMR—Nuclear Magnetic Resonance.

## 8. Techno-Economic Considerations for Industrial-Scale Keratinase Applications

Keratinases present a promising opportunity, an alternative to traditional chemical methods, for industrial waste recycling for environmental sustainability [100,193,194]. The economic viability of producing keratinases on a large scale is improved by using inexpensive keratinous waste as fermentation substrates, which helps lower production costs and reduce environmental waste [100]. Nonetheless, technological challenges remain due to the low enzyme yields from native keratinolytic organisms [195]. To boost keratinase production, molecular strategies like plasmid selection, promoter engineering, chromosomal integration, and codon optimization have been used to enhance keratinase production in heterologous expression systems such as *Escherichia coli*, *Bacillus* sp., and *Pichia pastoris* [195]. These methods aim to increase enzyme production to meet industrial demands and support sustainable development through green technology [100]. According to life cycle assessments (LCA), enzymatic dehairing of raw hides significantly diminishes environmental impacts when compared to chemical dehairing, largely due to decreased toxicant generation and energy consumption [196]. Transforming keratinous waste into valuable bioresource materials through biorefining reduces carbon footprint and supports the shift from a linear economy to a circular economy [197].

## 9. Limitations and Future Research Needs

Research on microbial transformation of keratinous biomass has been on an upward trajectory due to the prospects of this strategy in generating valuable products from waste bioresources. Among fungal strains, dermatophytes have been endowed with outstanding keratinolytic activity. However, this trait of dermatophytes has been linked with pathogenicity, allowing them to penetrate tissues, access nutrients, and establish infection. This observation has limited their commercial prospects. Studies on mining these important genes from the genome of keratinolytic fungi and expressing them in competent industrial hosts are lacking, suggesting new and promising avenues for research advancement. Most research focuses on the use of a single bacterial strain for the degradation of keratinous residues. The application of a single microbial strain presents certain limitations, such as producing keratinase with a narrow catalytic spectrum, leading to poor digestion of the keratin polymer. Recently, molecular optimization has been used to enhance microbial keratinase’s versatility and functional properties for the broader implementation of green technology in various industries. This protein engineering approach has created robust keratinolytic enzymes with improved performance. However, it does not provide a comprehensive solution as the environmental factors negatively impact the microbial strains due to the fluctuation of media conditions. Therefore, microbial consortia can be explored for industrial-scale degradation of keratinous waste due to the complexity and high stability of the system. A comprehensive understanding of microbial ecology through metagenomic sequencing and annotation, metabolic complementation, and synergistic interactions of the enzyme cocktails will offer insights into mechanisms that accelerate efficient microbial keratinolysis. Developing effective microbial consortia can also facilitate establishing efficient bioreactors for an industrial-scale keratinous waste biorefinery.

## 10. Conclusions

The review highlights the potential of microbial keratinases as an eco-friendly tool for valorizing keratinous waste biomass into valuable peptides. Keratinous wastes, including hair, chicken feathers, and horns, are generated in large quantities from various industries and accumulate in the environment due to their slow degradation caused by high cysteine disulfide bonds. Traditional methods of managing this waste have several disadvantages. Keratinases produced by bacteria, fungi, and actinomycetes can efficiently degrade the recalcitrant keratinous biomass into beneficial amino acids and peptides. These enzymes have multifaceted applications in cosmetics, organic fertilizers, leather treatment, animal feed, and detergents. Optimizing culture conditions can enhance keratinase production for industrial applications. The review emphasizes the need to explore more keratinolytic strains, improve keratinase yields, develop more robust strains and utilize microbial consortia to develop sustainable strategies for keratinous waste management and generate value-added products.

## Figures and Tables

**Table 1:** Different keratinolytic bacteria from diverse environmental sources.

Keratinolytic Bacteria	Source	Reference
*Bacillus licheniformis* PWD-1	Poultry waste	[45]
*Microbacterium* sp. kr10	Decomposing feathers	[65]
*Bacillus subtilis* S14	Soil	[63]
*Bacillus pseudofirmus*	Alkaline soda lake	[11]
*B. pumilus* AT16	Tunicate Didemnum maculosum	[66]
*B. subtilis* DB01	Harbour sediment	[66]
*Chryseobacterium indologenes* TKU014	Soil	[67]
*B. licheniformis* ER-15	Soil	[64]
*Streptomyces albidoflavus* K1-02	Hen house soil	[68]
*Chryseobacterium aquifrigidense* FANN1	Poultry dumpsites	[69]
*Bacillus macroides*	Dry meadow soil	[52]
*Bacillus cereus*	Dry meadow soil	[52]
*Chryseobacterium* sp. strain kr6	Poultry waste	[70]
*Microbacterium* sp. Kr10	Decomposing feathers	[47]
*Arthrobacter* sp. NFH5	Soil	[71]
*Meiothermus* sp. I40	Water from a hot spring	[72]
*Micromonospora* sp. AYA2000	Protoplast fusion	[73]
*Vibrio* sp. Kr2	Poultry abattoir soil	[1]
*Pseudomonas* sp. 3096-4	Decomposing wool	[74]
*Paracoccus* sp. WJ-98	Soil from a poultry factory	[75]
*Lysobacter* sp. NCIMB 9497	Collection culture	[76]
*Stenotrophomonas* sp.	Deer fur	[17]
*Thermoanaerobacter* *keratinophilus*	Geothermal hot spring	[77]
*Xanthomonas* *maltophila* POA-1	Poultry waste	[29]
*Fervidobacterium islandicum* AW-1	Geothermal hot stream	[60]

## Data Availability

No new data were created or analyzed in this study. Data sharing is not applicable to this article.
